# The association between leisure engagement and loneliness before and during the COVID-19 pandemic: A Nordic population-based study

**DOI:** 10.1177/14034948231171964

**Published:** 2023-05-10

**Authors:** Fredrica Nyqvist, Ingeborg Nilsson, Marina Näsman, Birgitta Olofsson

**Affiliations:** 1 Faculty of Education and Welfare Studies, Social Policy, Åbo Akademi University, Finland; 2 Department of Community Medicine and Rehabilitation, Occupational Therapy, Umeå University, Sweden; 3 Department of Nursing, Umeå University, Sweden; 4 Department of Surgical and Perioperative Science, Orthopaedics, Umeå University, Sweden

**Keywords:** Leisure engagement, loneliness, Sweden, Finland, COVID-19 pandemic

## Abstract

**Aim:**

The main aim of this study was to examine leisure engagement and loneliness among older adults before and during the COVID-19 pandemic by analysing population-based data from western Finland and northern Sweden.

**Methods:**

The data originated from the Gerontological Regional Database (GERDA) postal questionnaire study conducted in 2016 (*n*=7996) and 2021 (*n*=8148) among older adults aged 65, 70, 75, 80 and 85 years. Associations between loneliness and leisure engagement were analysed using logistic regression.

**Results:**

In total, 10% and 9% of the older adults reported loneliness in 2016 and 2021, respectively. The results showed that a lack of engagement in socialising and pleasure was independently associated with loneliness in both study years, while a lack of engagement in cultural activities was associated with loneliness in 2016 only. In 2021, the likelihood of experiencing loneliness was higher in the Finnish region than in the Swedish region. In addition, those reporting a decrease in hobby and socialising leisure activities due to the COVID-19 pandemic were more likely to report loneliness.

**Conclusions:**

**Most leisure activities decreased during the pandemic, suggesting an increase in social isolation. However, this did not reflect an increase in loneliness in the studied regions. The evidence suggests that leisure engagement, especially socialising activities, continued to be important for well-being among older adults during the pandemic. Further, loneliness was affected by contextual factors as well as individual-level characteristics. Thus, according to the measures reported here, the pandemic seemed to have a slightly weakened well-being impact in Finland.**

## Introduction

Loneliness in later life is a shared public health concern across the Nordic countries [1]. Although loneliness levels are lower in the Nordic countries than in other European countries, loneliness remains prevalent among older adults, ranging from 16.6% in Denmark to 23.3% in Finland and 23.6% in Sweden [1]. However, whether the COVID-19 pandemic has exacerbated loneliness levels among older adults remains contested. A systematic review by Su et al. [2] showed that the pooled prevalence of loneliness was higher in studies conducted during the pandemic than in pre-pandemic studies. However, longitudinal studies analysing data collected in Norway [3] and Sweden [4] revealed that the pandemic did not have a negative individual-level effect on loneliness. Since leisure engagement has often been used as an intervention strategy to prevent and alleviate loneliness in older adults [5], the pandemic, with imposed restrictions on social and public gatherings, added an extra layer of complexity to our understanding of leisure and loneliness, which calls for more research on leisure engagement and loneliness during challenging times.

Leisure can be conceptualised as an activity people choose freely to do [6] and as an activity done for relaxation, entertainment or personal development [7]. It is well known that engagement in leisure activities is related to health and well-being (including lower levels of loneliness) in older adults [8–10]. Leisure engagement can lead to meaningfulness, thereby easing the ability to cope with various health conditions and mental issues [11,12]. Being engaged in leisure activities also presents opportunities for social interaction and social support, which could promote mental health [13] and contribute to well-being [14]. The psychological mechanisms behind the relationship between leisure and well-being have been documented in the literature and include affiliation, autonomy, detachment, mastery and meaning as mediating factors [15]. With its restrictions, the pandemic presented profound threats to continuing leisure life and engagement, especially for older adults, with the potential to reduce well-being, including increased loneliness.

In order to reduce the spread of the virus during the COVID-19 pandemic, significant measures were taken, and the two Nordic countries included in this study (Sweden and Finland) implemented various policy measures in the early stage of the outbreak [16,17]. However, Sweden has been regarded as an outlier in relation to both responses and outcomes, including higher mortality rates [16,17]. For example, Finland implemented lockdown in the spring of 2020, whereas Sweden remained relatively open and chose to rely more on voluntary adherence to recommendations. The authorities in both countries, however, emphasised the need to protect children, older adults over the age of 70 and the chronically ill. For example, all kinds of leisure activities, including social gatherings, in Swedish nursing homes were banned for older adults [18] and highly restricted for all other older adults, including physical and social distancing [19]. It has been shown that in areas with social isolation and restrictions, an increased sedentary lifestyle was reported among older adults whose level of physical activity decreased [20]. Further, among both Swedish and Finnish seniors, a leisure-related adaptation strategy was identified [21,22]. They described a reorganisation of leisure time to build new routines and aimed to remain active and enjoy more passive or slow leisure activities such as watching TV, listening to the radio and reading. However, we know little about how the pandemic influenced leisure engagement and affected everyday living among older adults.

Therefore, in this study, we scrutinised various leisure engagement activities identified in previous research on older adults as important in understanding well-being [8–10]. The main aim of this study was to examine leisure engagement and loneliness among older adults before and during the COVID-19 pandemic by analysing population-based data in western Finland and northern Sweden. This aim was further divided into two subsidiary aims: to assess the association between leisure engagement and loneliness in 2016 and 2021 and the association between reported changes in leisure engagement due to the COVID-19 pandemic and loneliness in 2021.

## Methods

### Sample

The data were derived from the Gerontological Regional Database (GERDA) survey conducted in Västerbotten in Sweden and Österbotten in Finland during the last quarters of 2016 and 2021 [23]. In 2016, a questionnaire was sent out by post to every individual born in 1930, 1935, 1940, 1945 and 1950, except for those in the city of Vaasa (Finland), where every second individual was selected, and the cities of Skellefteå and Umeå (Sweden), where every third individual was selected. The 2021 survey was sent out during the so-called third wave of the pandemic, characterised by steep increases in cases in both Finland and Sweden (https://ourworldindata.org). The same sampling procedure as in 2016 was applied in 2021, with the additional inclusion of individuals born in 1955. The oldest age group in 2021 was excluded from the present analysis in order to obtain corresponding age groups from both study years, namely 65-, 70-, 75-, 80- and 85-year-olds. The participants were selected from Market Information in Sweden AB and the Digital and Population Data Services Agency in Finland. The response rates in Sweden were 70.8% in 2016 and 59.1% in 2021, whereas in Finland, they were 61.7% and 46.2%, respectively, in the two study waves. The sample of the current study consists of 7996 individuals from 2016 and 8148 individuals from 2021.

The GERDA survey data collection was approved by the Regional Ethical Review Board in Umeå, Sweden, in 2016 and the Swedish Ethical Review Authority in 2021 (2016-367–32 and 2021-04965). In Finland, ethical approval is not mandatory for anonymous population-based postal surveys (Medical Research Act 488/1999; the English translation is available at http://www.finlex.fi/en/laki/kaannokset/1999/en19990488).

### Measures

Loneliness was used as an outcome variable and was measured with the question ‘Do you suffer from loneliness?’ (yes/no). Leisure engagement in 2016 and 2021 was based on the Modified Norling Petterson Selander (MNPS) Interest Checklist [24] and was assessed with questions concerning whether the respondent took part in various leisure activities. For this study, we analysed the following activities: socialising (e.g. with family, friends), associational, pleasure (e.g. dancing, restaurants), cultural, hobby and religious activities (yes/no). In 2021, an additional question for each leisure activity was added, and the respondents were also asked whether their level of engagement in these activities had increased, decreased or experienced no change due to the COVID-19 pandemic.

The explanatory variables included age (65, 70, 75, 80 or 85), sex (male, female), living situation (alone, cohabiting), educational level (primary or lower secondary, upper secondary) and region (Västerbotten Sweden, Österbotten Finland). Making ends meet was assessed with the following question ‘In your economic situation, is it possible to make ends meet?’ The responses were categorised into yes (with some difficulty, with difficulty, with great difficulty) and no (without difficulty). Self-rated health was assessed with the question ‘In general, how would you say your health is?’ The responses were based on a five-point scale (excellent, very good, good, fair or poor). This variable was dichotomised into good health (excellent, very good or good) and poor health (fair or poor).

The missing values were high for the leisure engagement activities, ranging between 4% and 12% in 2016 and 8% and 17% in 2021 as opposed to the explanatory variables showing less than 3% missing values in both study years. The missing values for changes in leisure engagement activities due to COVID-19 ranged between 19% and 33%. Those who did not respond to the leisure engagement activities in 2016 were more likely to be living in Finland, were older, reported a lower educational level, lived alone and reported poor self-rated health (tested with the chi-square test, *p*<0.05). In 2021, the same pattern was observed for the missing values. However, a significant difference in non-response was observed for making ends meet. Thus, those with difficulties had more missing values. The pattern of non-response for sex was mixed in both study years and, in most cases, non-significant. Those with missing values were excluded from further analyses.

### Analysis

**Figure 1.**
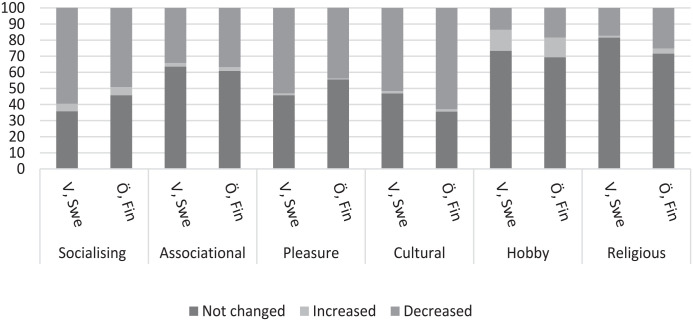
Share (%) of changes in leisure engagement due to the COVID-19 pandemic by region.

The distribution (%) of leisure engagement, loneliness and all explanatory variables is reported by study years (2016, 2021) and region (Sweden, Finland) in Table I, whereas the distribution (%) of changes in leisure engagement due to the COVID-19 pandemic is reported in Figure 1. Multivariable logistic regression analyses were conducted to identify factors associated with loneliness in 2016 and 2021, respectively (Tables II and III). First, the leisure engagement variables and region were entered one by one to test their association with loneliness (model 0). Next, all the leisure engagement variables and regions were entered in the same model (model 1). In the final model, all the variables were entered, including leisure engagement, region, age, sex, living situation, educational level, making ends meet and self-rated health (model 2). Finally, logistic regression analyses were repeated (model 0–model 2) for the variables assessing changes in leisure engagement due to the COVID-19 pandemic (Table IV). For this analysis, response categories recording increases (as opposed to decreases) and no change were grouped together. The data were analysed using the IBM SPSS Statistics for Windows v28 (IBM Corp., Armonk, NY).

**Table I.** Descriptive characteristics of the sample by study year and region (2016, *N*=7996; 2021, *N*=8148).

	2016			2021		
	Västerbotten, Sweden	Österbotten, Finland		Västerbotten, Sweden	Österbotten, Finland	
	% (*n*)	% (*n*)	*p*	% (*n*)	% (*n*)	*p*
Age groups (years)						
65	27.6 (1207)	30.1 (1084)		40.7 (2003)	23.8 (770)	
70	30.8 (1345)	30.9 (1114)		20.3 (997)	27.5 (887)	
75	19.8 (864)	16.3 (588)		21.7 (1067)	27.5 (888)	
80	13.7 (599)	14.3 (517)		10.4 (510)	12.4 (402)	
85	8.1 (356)	8.4 (302)	0.001	6.9 (341)	8.8 (283)	<0.001
Sex						
Male	48.6 (2123)	44.8 (1623)		49.9 (2406)	44.8 (1441)	
Female	51.4 (2249)	55.2 (1998)	<0.001	50.1 (2420)	55.2 (1776)	<0.001
Living situation						
Alone	29.9 (1288)	26.5 (953)		29.3 (1428)	24.8 (794)	
Cohabiting	70.1 (3021)	73.5 (2643)	<0.001	70.7 (3439)	75.2 (2408)	<0.001
Educational level						
Lower secondary	45.9 (1960)	38.6 (1384)		33.0 (1590)	38.3 (1229)	
Upper secondary	54.1 (2311)	61.4 (2204)	<0.001	67.0 (3227)	61.7 (1979)	<0.001
Difficulty in making ends meet						
Yes	34 (1452)	37.3 (1309)		32.3 (1554)	35.6 (1112)	
No	66 (2815)	62.7 (2201)	0.003	67.7 (3255)	64.4 (2010)	0.002
Self-rated health						
Poor	34 (1471)	38.2 (1371)		30.4 (1463)	30.1 (962)	
Good	66 (2855)	61.8 (2218)	<0.001	69.6 (3353)	69.9 (2235)	0.747
Loneliness						
Yes	10.3 (433)	9.5 (328)		8.6 (410)	9.5 (298)	
No	89.7 (3756)	90.5 (3114)	0.242	91.4 (4364)	90.5 (2823)	0.133
*Leisure engagement*						
Socialising						
Yes	97.3 (4116)	97.4 (3331)		96.3 (4523)	97 (2766)	
No	2.7 (113)	2.6 (89)	0.850	3.2 (150)	3 (86)	0.639
Association						
Yes	45.6 (1804)	45.6 (1360)		41.5 (1846)	42.9 (1107)	
No	54.5 (2151)	54.4 (1620)	0.984	58.5 (2601)	57.1 (1473)	0.267
Pleasure						
Yes	49.4 (1974)	33 (991)		49.3 (2192)	30.1 (749)	
No	50.6 (2025)	67 (2016)	<0.001	50.7 (2253)	69.9 (1736)	<0.001
Cultural						
Yes	87.5 (3585)	91.9 (3060)		49.4 (2117)	61 (1489)	
No	12.5 (513)	8.1 (269)	<0.001	50.6 (2172)	39 (952)	<0.001
Hobby						
Yes	63.1 (2560)	70.5 (2244)		65.8 (2895)	63.5 (1613)	
No	36.9 (1496)	29.5 (940)	<0.001	34.2 (1520)	36.5 (927)	0.057
Religious						
Yes	20.8 (823)	36 (1094)		16.7 (742)	30.9 (789)	
No	79.2 (3127)	64 (1945)	<0.001	83.3 (3694)	69.1 (1767)	<0.001

**Table II.** Odds ratios (ORs) and 95% confidence intervals (CIs) for the probability of feeling loneliness by leisure engagement and region in 2016.

	Model 0		Model 1 (*N*=5970)		Model 2 (*N*=5732)	
	OR	95% CI	OR	95% CI	OR	95% CI
*Leisure engagement*						
Socialising						
Yes	1		1		1	
No	4.68***	(3.42–6.42)	3.77***	(2.65–5.38)	2.80***	(1.89–4.16)
Association						
Yes	1		1		1	
No	1.45***	(1.22–1.71)	1.30**	(1.07–1.58)	1.23	(0.99–1.51)
Pleasure						
Yes	1		1		1	
No	1.89***	(1.59–2.26)	1.80***	(1.48–2.20)	1.31*	(1.06–1.63)
Cultural						
Yes	1		1		1	
No	1.71***	(1.37–2.14)	1.19	(0.92–1.54)	1.33*	(1.00–1.77)
Hobby						
Yes	1		1		1	
No	1.20*	(1.02–1.42)	0.96	(0.79–1.16)	0.88	(0.72–1.08)
Religious						
Yes	1		1		1	
No	0.85	(0.71–1.01)	0.72**	(0.59–0.89)	0.87	(0.69–1.09)
Region						
Österbotten, Finland	1		1		1	
Västerbotten, Sweden	1.09	(0.94–1.27)	1.24*	(1.03–1.50)	1.16	(0.95–1.41)
Age groups (years)						
65					1	
70					0.91	(0.70–1.17)
75					1.09	(0.82–1.46)
80					1.21	(0.88–1.64)
85					1.17	(0.81–1.71)
Sex						
Male					1	
Female					1.43***	(1.16–1.76)
Living situation						
Cohabiting					1	
Alone					3.11***	(2.56–3.79)
Educational level						
Lower secondary					0.88	(0.72–1.08)
Upper secondary					1	
Difficulty in making ends meet						
No					1	
Yes					1.50***	(1.24–1.82)
Self-rated health						
Good					1	
Poor					2.40***	(1.97–2.93)

* *p*<0.05; **p<0.01; ***p<0.001.

**Table III.** Odds ratios (ORs) and 95% confidence intervals (CIs) for the probability of feeling loneliness by leisure engagement and region in 2021.

	Model 0		Model 1 (*N*=5945)		Model 2 (*N*=5737)	
	OR	95% CI	OR	95% CI	OR	95% CI
*Leisure engagement*						
Socialising						
Yes	1		1		1	
No	4.02***	(2.95–5.49)	3.15***	(2.22–4.70)	2.13***	(1.42–3.15)
Association						
Yes	1		1		1	
No	1.43***	(1.20–1.71)	1.23	(0.99–1.52)	1.24	(0.98–1.57)
Pleasure						
Yes	1		1		1	
No	1.97***	(1.63–2.37)	1.62***	(1.30–2.01)	1.29*	(1.02–1.63)
Cultural						
Yes	1		1		1	
No	1.53***	(1.28–1.82)	1.24*	(1.01–1.53)	1.20	(0.96–1.52)
Hobby						
Yes	1		1		1	
No	1.46***	(1.23–1.73)	1.17	(0.97–1.43)	1.04	(0.84–1.28)
Religious						
Yes	1		1		1	
No	0.90	(0.74–1.10)	0.83	(0.65–1.06)	0.93	(0.72–1.22)
Region						
Österbotten, Finland	1		1		1	
Västerbotten, Sweden	1.12	(0.96–1.32)	0.97	(0.79–1.19)	0.76*	(0.62–0.96)
Age groups (years)						
65					1	
70					1.09	(0.83–1.44)
75					0.86	(0.65–1.14)
80					1.16	(0.83–1.64)
85					1.38	(0.94–2.01)
Sex						
Male					1	
Female					1.27*	(1.03–1.57)
Living situation						
Cohabiting					1	
Alone					4.26***	(3.45–5.24)
Educational level						
Lower secondary					0.79*	(0.63–0.98)
Upper secondary					1	
Difficulty in making ends meet						
No					1	
Yes					1.68***	(1.36–2.06)
Self-rated health						
Good					1	
Poor					2.08***	(1.68–2.57)

* *p*<0.05; **p<0.01; ***p<0.001.

**Table IV.** Odds ratios (ORs) and 95% confidence intervals (CIs) for the probability of feeling loneliness by changes in leisure engagement due to the pandemic.

	Model 0		Model 1 (*N*=4559)		Model 2 (*N*=4421)	
	OR	95% CI	OR	95% CI	OR	95% CI
*Changes in leisure engagement*						
Socialising						
No change, increased	1		1		1	
Decreased	1.17	(0.98–1.40)	1.22	(0.97–2.56)	1.31*	(1.02–1.68)
Association						
No change, increased	1		1		1	
Decreased	1.14	(0.94–1.38)	0.99	(0.77–1.29)	0.91	(0.69–1.21)
Pleasure						
No change, increased	1		1		1	
Decreased	1.09	(0.99–1.20)	0.79*	(0.62–0.99)	0.93	(0.72–1.21)
Cultural						
No change, increased	1		1		1	
Decreased	1.05	(0.96–1.15)	0.75*	(0.59–0.96)	0.87	(0.67–1.14)
Hobby						
No change, increased	1		1		1	
Decreased	1.94***	(1.55–2.41)	1.95***	(1.47–2.57)	1.51**	(1.12–2.05)
Religious						
No change, increased	1		1		1	
Decreased	0.81***	(0.73–0.91)	1.40*	(1.06–1.86)	1.28	(0.95–1.74)
Region						
Österbotten, Finland	1		1		1	
Västerbotten, Sweden	0.89	(0.76–1.04)	0.84	(0.67–1.05)	0.69**	(0.54–0.88)
Age groups (years)						
65					1	
70					1.15	(0.85–1.56)
75					0.00	(0.65–1.22)
80					1.32	(0.90–1.94)
85					1.13	(0.72–1.78)
Sex						
Male					1	
Female					1.21	(0.95–1.53)
Living situation						
Cohabiting					1	
Alone					4.33***	(3.43–5.48)
Educational level						
Lower secondary					1.02	(0.80–1.31)
Upper secondary					1	
Difficulty in making ends meet						
No					1	
Yes					1.76***	(1.40–2.22)
Self-rated health						
Good					1	
Poor					2.49***	(1.97–3.15)

* *p*<0.05; **p<0.01; ***p<0.001.

## Results

Descriptive information regarding the study year and region is provided in Table I. In total, 10% of the respondents reported loneliness in 2016, and 9% reported loneliness in 2021. The share of older adults who were active in leisure engagement was generally lower in 2021 than in 2016, with the exception of hobby activities in Sweden.

Descriptive information regarding reported changes in leisure engagement due to the COVID-19 pandemic is shown in Figure 1. A decrease was reported in all activities, most notably in pleasure, cultural and socialising activities.

The logistic regression models in Table II show the likelihood of experiencing loneliness in 2016 by leisure engagement, region and various explanatory variables. According to model 0, those who were not actively engaged in socialising, associational, pleasure, cultural and hobby activities were more likely to experience loneliness. Furthermore, when all leisure activities and region were included in model 1, hobby and cultural activities lost their significance, whereas the likelihood of loneliness was higher for those actively engaged in religious activities. Further, the Swedish region was also associated with a higher likelihood of experiencing loneliness. In model 2, when all the variables were controlled for, not being actively engaged in socialising, pleasure and cultural leisure activities was associated with loneliness. The relationship between region and loneliness was no longer statistically significant, whereas being female, living alone, difficulty making ends meet and poor self-rated health were independently associated with loneliness in 2016.

Table III shows the likelihood of experiencing loneliness in 2021. In the univariate analyses, (model 0), those who were not actively engaged in socialising, associational, pleasure, cultural and hobby activities were more likely to report loneliness. In model 1, hobby and associational activities lost their significance. In model 2, lack of socialising and pleasure activities were independently associated with loneliness. The odds ratio for loneliness was significantly lower for Sweden compared to Finland. Finally, female sex, living alone, difficulty making ends meet, higher educational level and poor self-rated health were significantly associated with loneliness.

A decrease in hobby activities and no change/an increase in religious engagement due to the COVID-19 pandemic were significantly associated with loneliness in model 0 (see Table IV). In model 1, no change/increase in pleasure and cultural leisure engagement increased the odds ratios of experiencing loneliness, whilst a decrease in hobby activities and religious engagement were associated with a higher likelihood of experiencing loneliness. In model 2, which included all the studied variables, a decrease in socialising and hobby leisure engagement were associated with loneliness. Further, living in the Finnish region, living alone, difficulty making ends meet and poor self-rated health increased the odds of experiencing loneliness.

## Discussion

The results revealed that a total of 10% and 9% of the older adults reported loneliness in 2016 and 2021, respectively. Our analyses showed that not being actively engaged in socialising and pleasure activities were independently associated with loneliness in both study years. However, no engagement in cultural activities was associated with loneliness in 2016 only. In 2021, the likelihood of reporting loneliness was higher in the Finnish region compared to the Swedish region. In addition, those reporting a decrease in hobby and socialising activities due to the COVID-19 pandemic were more likely to report loneliness.

Leisure engagement is considered an important means to cope with stress and social isolation. It seems reasonable to assume that social distancing and staying at home hindered older adults’ leisure engagement and that this increased social isolation and loneliness. Our data showed that the lack of engagement of older adults in socialising or pleasure activities was associated with loneliness both before and during the pandemic, which could mean that the studied group of older adults regarded these activities as particularly meaningful and valuable for their health and well-being [8–10]. As expected, a lower proportion of older adults were engaged in various leisure activities in 2021. However, this was not reflected in an increase in loneliness on a population level.

It is worth noting that many older adults were active in 2021, despite various restrictions aimed at curbing the transmission of the coronavirus [16,17]. One reason might be related to the timing of our data collection, which began in the early phase of the third wave of the pandemic, and that social-distancing strategies were less strict in the mid-autumn of 2021. The timing of data collection during the pandemic related to the findings is an issue discussed by, for example, Gustafsson et al. [19]. Further, it could be related to adaptation and new ways of performing different leisure activities [22], including small-group and online leisure events and outdoor social gatherings [25]. Others described a more sedentary lifestyle indoors, that is, at home [21,22].

Cultural activities were performed less frequently in 2021 compared to 2016. The cultural sector was affected by severe restrictions during the pandemic, with the closure of theatres, cinemas, museums and music events. Nevertheless, there was no statistically significant association between cultural activities and loneliness in 2021. Instead, those who reported a decrease in hobby activities were more likely to be lonely. Hobby activities can be performed alone as well as in groups, and although we have no data regarding the type of hobby activities performed, renewed interest in and adaptation to at-home hobbies have been reported during the COVID-19 pandemic [26]. Similarly, another study reported a decrease in cultural activities but increased engagement in hobbies [27]. Engagement in religious activities differed between the studied regions, and it was more common in the Finnish region to be engaged in religious activities in both study years. The association between religious activities and loneliness was the reverse of other activities. Thus, the likelihood of reporting loneliness was higher in the active group, albeit at a statistically non-significant level. Religiosity has previously been associated with both positive and negative feelings. Church attendance and other religious activities arguably increase social support, decrease social isolation and thus have positive well-being effects [28], whereas religious struggles have been linked to social isolation, depression and loneliness [29].

On a population level, we observed similar levels of loneliness before and during the pandemic. This corroborates some findings [3,4] and contradicts others pointing to an increase in loneliness, particularly among those who were most at risk of social isolation and loneliness, including those receiving home care [18,19]. It is noteworthy that in the analysis of region as an explanatory factor in the likelihood of experiencing loneliness in 2021, Finland scored higher. In response to the COVID-19 pandemic, Sweden and Finland imposed several measures to prevent COVID-19, with different levels of intensity and varying recommendations and legislative measures [16,17]. Although loneliness is highly influenced by individual-level characteristics and resources, it is also affected by policy measures and contextual features, including for example how loneliness is presented in the public discourse, potentially explaining the observed regional differences in 2021 [1]. Future research should focus more on policy and the structural impacts on loneliness, a research field that is still relatively unexplored.

### Limitations

The share of lonely older adults was higher in previous research than in the present context, including in Finland and Sweden [1]. However, it should be noted that we assessed the experience of suffering from loneliness instead of frequent loneliness. In our study, loneliness was examined by a direct single-item loneliness question with a yes/no answer. Further, the use of a single-item question cannot discriminate between social and emotional loneliness, as suggested by Weiss [30]. Consequently, any comparison of the results with other loneliness studies should be attempted with caution.

Missing data on leisure engagement also posed a limitation, and the results might be biased, since more resourceful older adults chose to answer the leisure engagement items. Although the response rate was relatively high, there was a risk of non-response bias, especially in the Finnish sample, which recorded a lower response rate in both study years. The reason for regional differences in the response rate is unknown. However, the response pattern resembled that of earlier waves conducted in 2005 and 2010. It could further be expected that those participating in the survey were somewhat healthier and less lonely than those who did not participate. Finally, our results are based on repeated population-based cross-sectional data, implying that we could not draw conclusions on either the direction of the relationships between leisure engagement and loneliness or the causes of differences found in leisure engagement.

## Conclusions

Most leisure activities, especially cultural and socialising activities, decreased during the pandemic, suggesting an increase in social isolation. However, this was not reflected in an increase in loneliness in our studied regions. Further, the evidence in our study suggests that leisure engagement, especially socialising activities, continued to be important for well-being among older adults during the pandemic. We also showed that loneliness was affected by contextual factors as well as individual-level characteristics. Thus, according to the measures reported here, the pandemic seemed to have a slightly weakened well-being impact in Finland.
